# Replacing red and processed meat, poultry, or fish with legumes and the risk of gallbladder diseases in a large British cohort

**DOI:** 10.1007/s00394-025-03828-1

**Published:** 2025-11-12

**Authors:** Fie Langmann, Daniel B. Ibsen, Luke W. Johnston, Aurora Perez-Cornago, Christina C. Dahm

**Affiliations:** 1 Department of Public Health, Aarhus University, Bartholins Allé 2, 8000 Aarhus C, Denmark; 2 Steno Diabetes Center Aarhus, Aarhus University Hospital, Palle Juul-Jensens, Boulevard 11, 8200 Aarhus C, Denmark; 3 European Commission, Joint Research Centre (JRC), Ispra, Italy

**Keywords:** Gallbladder disease, Gallstone, Legumes, Red and processed meat

## Abstract

**Purpose:**

Legumes are promoted as climate-friendly and healthy protein sources. This study evaluated the association between replacing red and processed meat, poultry, or fish with equal amounts of legumes and the risk of gallbladder disease.

**Methods:**

Participants from the UK Biobank Cohort who completed two or more 24 h dietary assessments and had complete information on covariates were included. Information on age, sex, and socioeconomic and lifestyle factors were collected at recruitment, while information on dietary intake was collected using multiple 24 h dietary assessments. Information on incident gallbladder disease (defined as cholelithiasis, cholecystectomy, or cholecystitis) was collected from health registries based on ICD10-diagnosis or operation codes. The rate of developing gallbladder diseases when replacing red and processed meat, poultry, or fish with legumes was estimated using multivariable-adjusted Cox Proportional Hazards regression analyses adjusted for potential confounders.

**Results:**

Over a median follow-up time of 10.5 (interquartile range: 10.4–10.9) years, 121,593 eligible participants provided 1,246,913 person-years of follow-up during which 3772 individuals developed gallbladder disease. Replacing 80 g/week of red and processed meat with legumes was associated with a lower rate of gallbladder disease (HR: 0.97, 95% CI 0.95; 0.98, *p* < 0.001). No association was found when replacing poultry or fish with legumes. Adjusting for BMI did not change the magnitude or direction of associations.

**Conclusions:**

Replacing red and processed meat with legumes was associated with lower rates of gallbladder disease. Further research in populations with higher legume intake is warranted to confirm these findings.

**Supplementary Information:**

The online version contains supplementary material available at 10.1007/s00394-025-03828-1.

## Introduction

Since 1990, global gallstone incidence and related comorbidities have risen by nearly 60%, with incidence rates up to 246/100,000 in Europe [1–5]. Gallbladder diseases (GBD) such as cholelithiasis (gallstone), cholecystitis (gallbladder inflammation), and cholecystectomy (removal of the gallbladder) are common, with global prevalences between 5 and 20% [3, 5, 6]. Cholelithiasis are primarily caused by excess cholesterol in the gallbladder, and risk factors include older age, elevated BMI, and female sex [1]. Moreover, Western dietary patterns high in energy, red and processed meat, and fats, and low in dietary fibre from vegetables and fruits have been associated with increased GBD risk [7, 8]. Western dietary patterns promote excess lipid storage in the liver, which may increase the risk of supersaturating the bile with cholesterol, causing cholesterol gallstone formation [2, 7, 9]. Studies have indicated that even low consumption of red and processed meats and poultry as individual dietary components increase the risk of GBD [10, 11].

Legume consumption in the range of 50–100 g/day is increasingly being promoted across dietary guidelines as a replacement for animal-based foods [12–14]. Compared to red and processed meat, legumes are lower in saturated fat, higher in dietary fibre, and have lower environmental impact, making them cornerstones in healthy and environmentally sustainable diets [13, 15, 16]. Yet, the health impact of high intakes of legumes is understudied in humans [17]. A recent review highlighted the sparsity and poor methodological quality of studies investigating the association between legume consumption and GBD [18]. The limited research in Western countries is likely due to the negligible consumption of legumes to date [15, 16, 19].

Identifying modifiable risk factors for GBD is central to mitigate the rising prevalences. This study aimed to investigate the association between replacing red and processed meat, poultry, or fish with legumes and the risk of GBD (cholelithiasis, cholecystitis, and cholecystectomy) in a UK population.

## Methods

### Study population and setting

The UK Biobank study recruited approximately 500,000 UK citizens aged 37–73 years between 2006 and 2010 in England, Scotland, and Wales [20]. At baseline, participants self-reported information on sociodemographic, physical, lifestyle, diet, and health-related characteristics through touch-screen questionnaires and a computer-assisted personal interview [20]. Trained professionals conducted standardised physical, anthropometric, and biomedical measurements [20].

All participants gave written, informed consent to participate prior to study entry. The UK Biobank was approved by relevant ethical authorities and conducted according to the Declaration of Helsinki.

### Assessment of diet

The UK Biobank assessed dietary intake using the Oxford WebQ, a self-administered, internet-based tool for 24 h dietary assessments [21–23]. The questionnaire comprised 206 food items and 32 beverages carefully selected using UK National Diet and Nutrition Survey data to reflect the most commonly consumed items in the UK adult population together with a pilot study in this population [23]. Portion sizes were assigned based on the way each question was phrased, using UK standard portion sizes and product information from UK online supermarkets. Grams of food consumed were calculated by multiplying the reported frequency consumed by the portion sizes [24]. It included help text, illustrative images, and food examples to guide participants. This questionnaire has been compared against interviewer-administered 24 h dietary recalls and validated biomarkers. The Oxford WebQ has been validated against recovery biomarkers for energy, protein and potassium, and was considered to perform well in approximating true dietary intake. This questionnaire also provided similar mean estimates of energy and nutrient intakes when compared with an interviewer administered 24 h dietary recall [22]. The tool was recently updated with new dietary variables [22].

Participants recruited from April 2009 to September 2010 completed their first Oxford WebQ at the assessment centre (N = 70,747). Those recruited before April 2009 were invited to complete the Oxford WebQ online, if they had provided a valid e-mail address. Invitations to complete the Oxford WebQ online were sent to all participants with valid e-mail addresses on four separate occasions (February 2011–June 2012) [25]. The Oxford WebQ was completed by 210,950 participants at least once. In this study, we included participants with at least two 24 h dietary assessments to estimate usual intake of food groups. Assessments were completed a mean of 21 weeks (SD: 5 weeks) apart.

Consumption of legumes, red and processed meats, poultry, fish, and other food groups (defined in Supplementary Table 1) were determined from the average daily food intakes of  ≥ two (maximum of five) 24 h dietary assessments to minimise the effects of random error and within-person variability [26], after which intakes were rescaled to g/week.

### Gallbladder diseases

Incident GBD cases included cholelithiasis, cholecystitis, and cholecystectomy assessed through linkage to the NHS registries. These registries follow strict documentation standards to ensure the accurate recording of events and are generally regarded as valid and reliable for capturing hospital-based diagnoses and events [27]. Continuous data quality checks and generation of data quality reports are conducted to maintain and ensure the accuracy of the reported hospital inpatient data [28]. Diagnoses were coded using the International Classification of Diseases and Related Health Problems 10th edition (ICD-10) or operative procedure codes (OPCS) [29]. Diagnosis of cholecystitis and cholelithiasis does not provide information on whether the patient recovers completely. Individuals might experience symptoms due to gallstones already present in the gallbladder without developing new gallstones. Individuals can develop both conditions more than once and have undiagnosed gallstones without symptoms [30]. We only used the first hospital inpatient record of incident cholecystitis or cholelithiasis. For cholelithiasis, ICD-10 codes were K80.0-K80.8 and OPCS codes were J21.1, J24.2–3, J26.1, J33.1–2, J41.1, J41.3, J49.1–2, and J52.1. For cholecystitis, ICD-10 codes were K81.0-K81.9. For cholecystectomy, OPCS codes were J18.1–3 and J18.8–9 [29].

### Covariates

We chose covariates a priori based on a review of the literature and directed acyclic graphs (Supplementary Fig. 1). Covariates included all other food group intakes, sex, age, ethnicity, yearly income, educational level, region of recruitment, cohabitation, physical activity, smoking status, history of gallbladder related conditions (diabetes, elevated cholesterol, hepatitis, cirrhosis), ever using hormone replacement therapy drugs (HRT) or oral contraceptives for women, number of pregnancies for women, or family history of diabetes, which were all self-reported. While rapid weight loss seems to increase risk of GBD [7], this information was not available so we used participants’ self-report of recent weight change in the past year assessed through the question: “Compared with one year ago, has your weight changed?” as an indicator for recent weight loss. Other covariates were Townsend Deprivation Score (positive values indicate higher deprivation; negative values indicate relative affluence [31]), obesity (BMI  ≥  30 kg/m^2^), and total serum bilirubin [32] measured at baseline by professionally trained staff.

### Exclusion criteria

We excluded participants who completed ≤ 1 Oxford WebQ or underwent cholecystectomy or were diagnosed with cholelithiasis or cholecystitis before the last completed Oxford WebQ. Missing data on covariates at baseline also resulted in exclusion, apart from missing information on serum bilirubin level. Some baseline blood sample assays returned errors with values outside the reportable range or aliquot problems, and 7119 participants had unknown total serum bilirubin concentrations [33]. If bilirubin concentrations were missing, they were coded as “unknown” rather than excluding the participant.

### Statistical analyses

We performed standard summary statistics and multi-variable adjusted Cox proportional hazards regression models to estimate the hazard ratios (HR) and corresponding 95% confidence intervals (CI) for GBD based on replacing red and processed meat, poultry, or fish with legumes. The substitution magnitude represented a weekly substitution where one serving of legumes or pulses (80 g) [34] replaced an equal amount of red and processed meat, poultry, or fish. The intake of all other food components (g/week) was held constant in the substitution models. Substitutions were modelled using the leave-one-out approach:

$$ \begin{aligned} log\left( {h\left( {t;x} \right)} \right) & = log\left( {h\left( {0;t} \right)} \right) + \beta_{1} Legumes\left( {80g/week} \right) \\ & \quad + \beta_{2} Totalfoodintake\left( {g/week} \right) \\ & \quad + \beta_{3} ^{\prime}Otherfoodgroups\left( {g/week} \right) \\ & \quad + \beta_{4} ^{\prime}Covariates \\ \end{aligned} $$

Apostrophes indicate a group of coefficients consisting of several individual variables (β3’ and β4’). Variables for intakes of each food group (β3’) and total food intake (β2) were included and held stable, while the food group that were to be substituted was left out of the model [35]. The estimated HR expresses the rate of GBD when keeping the total food intake stable while specifying that 80 g/week of food should come from legumes instead of the left-out component (red and processed meat, poultry, or fish).

We used age as the underlying time scale in the analyses and calculated person-years at risk from the date of last completed Oxford WebQ to the date of death, loss to follow-up, diagnosis of GBD, or right censoring, whichever occurred first. Participants were right censored on the most recent full follow-up for the outcomes (October 31st, 2022). The proportional hazards assumption was evaluated as satisfied using Schoenfeld residuals. The linearity assumption for substitution analysis was assessed using likelihood ratio tests comparing exposures modelled as linear predictors and restricted cubic splines at the 10th, 50th, and 90th percentiles [36]. The test indicated no significant improvements in the model fit when modelling the exposure with splines instead of linear predictors.

In Model 1, we stratified by age (< 45, 45–49, 50–54, 55–59, 60–64,  ≤ 65 years) at recruitment, sex (male, female), and geographical recruitment region (ten UK regions), and adjusted for total food intake (g/week) and intakes of all other food groups, except for the two substitutes. In Model 2, we further adjusted for ethnicity (White or other), socioeconomy (Townsend deprivation score [continuous], educational level [low: Certificate of Secondary Education (CSE), National Vocational Qualifications, Higher National Diploma, Higher National Certificates, other professional qualifications, or equivalent; intermediate: A levels, O levels, General Certificate of Secondary Education, or equivalent; high: College or University degree], yearly income [<  18,000£, 18,000–30,999£, 31,000–51,999£, 52,000–100,000£,  >  100,000£, unknown], and cohabitation [alone, with spouse or partner, with non-partner, unknown]), physical activity (low [≤ 918 METs/week], moderate [≥ 918–3706 METs/week], high [≤ 37,069 METs/week], unknown), smoking status (never, former, current < 15 cigarettes per day, current  ≥ 15 cigarettes per day, unknown), recent weight loss (yes, no), serum bilirubin levels (normal: [<  21 µmol/L], elevated [≥ 21 µmol/L] [37], unknown), previous diagnosis of diabetes, elevated cholesterol, hepatitis, or cirrhosis (yes, no), use of hormonal drugs for women (HRT, oral contraceptives [yes, no]), number of pregnancies for women (continuous), and family history of diabetes (yes, no). As obesity may both confound and mediate the association, we further adjusted for BMI  ≥  30 kg/m^2^ (yes, no) in model 3.

### Secondary and sensitivity analyses

Secondary analyses included: (i) main analyses restricted to consumers of legumes (N = 49,505); (ii) modelling legume consumption as an 80 g/week higher legume consumption among consumers of legumes (N = 49,505) without substitutions as this analysis did not adjust for total food intake; (iii) in the total cohort, running cholelithiasis, cholecystectomy, and cholecystitis as separate outcomes; (iv) stratified analyses by (a) tertiles of age (< 53, 53–61, > 61 years), (b) sex (male, female), and (c) BMI categories (< 25, 25–29.9, ≥ 30 kg/m^2^) to explore potential effect modification.

For sensitivity analyses we (i) included fresh peas in the legume exposure; (ii) excluded soy milk consumption from the legume exposure; (iii) removed participants with fewer than three completed Oxford WebQs; (iv) removed participants with unknown or highest (≥ 90th percentile) serum bilirubin concentrations [32]; (v) analysed red and processed meat separately; (vi) removed participants with history of diabetes, elevated cholesterol, hepatitis, or cirrhosis. Adjustments for secondary and sensitivity analyses matched Model 2.

We ran all analyses in R (Version 4.1.1, The R Foundation for Statistical Computing) with a significance level of 5%.

## Results

**Fig. 1:**
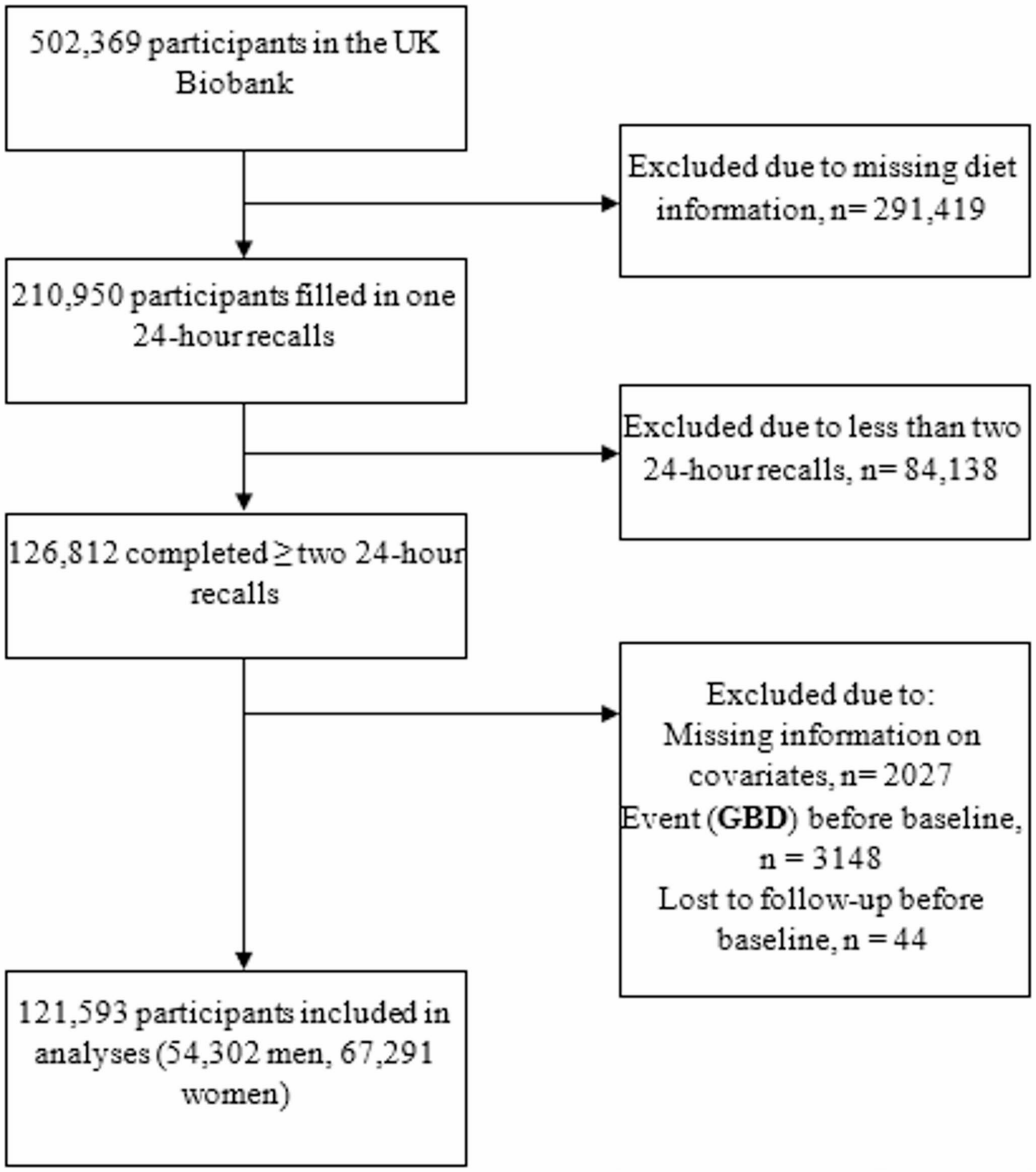
Flowchart of participants in the UK Biobank eligible for inclusion

Among the 502,369 participants in the UK Biobank prospective cohort, 126,812 completed ≥ 2 Oxford WebQs. Of these, 2027 were excluded due to missing covariate data, 3148 had an incident event before the start of follow-up, and 44 were lost to follow-up before baseline (last completed Oxford WebQ) (Fig. 1). During a median follow-up time of 10.5 (interquartile range: 10.4–10.9) years, 121,593 individuals (54,302 men, 67,291 women) contributed 1,246,913 person-years of follow-up, during which 3772 GBD incidents occurred.

A large proportion of participants (N = 72,088) reported no legume consumption. Those with the highest legume intake had significantly lower consumption of animal-based foods such as red and processed meats, poultry, and fish compared to the full sample and individuals with lower legume intake (Table 1). Those who developed GBD had lower deprivation, higher BMI, and were more likely to smoke, report a recent weight loss, and having used HRT drugs or oral contraceptives compared to the full sample (Supplementary Table 2).

**Table 1:** Baseline characteristics in the UK biobank cohort across legume consumption strata (N = 121,593)

		Legume consumption strata					
Characteristics	All participants	Non-consumers (0 g legumes/week)		Low (< 163 g legumes /week)	Medium (163–358 g legumes /week)	High (> 358 g legumes /week)	
	N = 121,593	N = 72,088		N = 15,262	N = 17,706	N = 16,537	
Legume consumption^a^, g/week	0 (0, 473)	0 (0, 0)		88 (41, 146)	245 (163, 315)	591 (420, 1,531)	
Gallbladder disease	3772 (3.1%)	2295 (3.2%)		431 (2.8%)	551 (3.1%)	495 (3.0%)	
Sex, female	67,291 (55%)	39,949 (55%)		8821 (58%)	9340 (53%)	9181 (56%)	
Age, years	57.0 (44.0, 66.0)	57.0 (45.0, 66.0)		57.0 (44.0, 66.0)	57.0 (44.0, 66.0)	56.0 (44.0, 66.0)	
*Yearly income £*							
> 100,000	8808 (7.2%)	5255 (7.3%)		1358 (8.9%)	1268 (7.2%)	927 (5.6%)	
52,000–100,000	28,353 (23%)	16,757 (23%)		3844 (25%)	4205 (24%)	3547 (21%)	
31,000–51,999	32,116 (26%)	18,991 (26%)		3998 (26%)	4672 (26%)	4455 (27%)	
18,000–30,999	26,147 (22%)	15,559 (22%)		3067 (20%)	3859 (22%)	3662 (22%)	
< 18,000	15,089 (12%)	8805 (12%)		1684 (11%)	2185 (12%)	2415 (15%)	
Unknown	11,080 (9.1%)	6721 (9.3%)		1311 (8.6%)	1517 (8.6%)	1531 (9.3%)	
*Educational level* ^*b*^							
High	57,341 (47%)	32,838 (46%)		8137 (53%)	8639 (49%)	7727 (47%)	
Intermediate	40,409 (33%)	24,797 (34%)		4552 (30%)	5647 (32%)	5413 (33%)	
Low	23,843 (20%)	14,453 (20%)		2573 (17%)	3420 (19%)	3397 (21%)	
Deprivation^c^	− 2.4 (− 4.7, 2.6)	− 2.5 (− 4.7, 2.4)		− 2.3 (− 4.6, 2.7)	− 2.3 (− 4.6, 2.8)	− 2.1 (− 4.6, 3.0)	
*Cohabitation*							
Alone	21,654 (18%)	12,616 (18%)		2627 (17%)	3139 (18%)	3272 (20%)	
With spouse/partner	90,575 (74%)	53,978 (75%)		11,481 (75%)	13,220 (75%)	11,896 (72%)	
Other non-partner	9162 (7.5%)	5393 (7.5%)		1122 (7.4%)	1320 (7.5%)	1327 (8.0%)	
Unknown	202 (0.2%)	101 (0.1%)		32 (0.2%)	27 (0.2%)	42 (0.3%)	
*Ethnicity*							
White	117,375 (97%)	70,155 (97%)		14,572 (95%)	16,931 (96%)	15,717 (95%)	
Other	4218 (3.5%)	1933 (2.7%)		690 (4.5%)	775 (4.4%)	820 (5.0%)	
*Physical activity* ^*d*^							
High	21,233 (17%)	12,176 (17%)		2,655 (17%)	3162 (18%)	3240 (20%)	
Moderate	53,952 (44%)	31,540 (44%)		6956 (46%)	8016 (45%)	7440 (45%)	
Low	29,404 (24%)	17,964 (25%)		3644 (24%)	4176 (24%)	3620 (22%)	
Unknown	17,004 (14%)	10,408 (14%)		2007 (13%)	2352 (13%)	2237 (14%)	
*Smoking status*							
Current, > 15 cigarettes/day	1756 (1.4%)	1145 (1.6%)		191 (1.3%)	230 (1.3%)	190 (1.1%)	
Current, < 15 cigarettes/day	3304 (2.7%)	2019 (2.8%)		391 (2.6%)	470 (2.7%)	424 (2.6%)	
Former	43,410 (36%)	25,323 (35%)		5501 (36%)	6497 (37%)	6089 (37%)	
Never	69,498 (57%)	41,497 (58%)		8734 (57%)	9953 (56%)	9314 (56%)	
Unknown	3625 (3.0%)	2104 (2.9%)		445 (2.9%)	556 (3.1%)	520 (3.1%)	
*Anthropometry*							
Weight loss past year^e^	18,310 (15%)	10,674 (15%)		2363 (15%)	2685 (15%)	2588 (16%)	
BMI ≥ 30 kg/m^2^	23,583 (19%)	14,286 (20%)		2760 (18%)	3450 (19%)	3087 (19%)	
*Female reproductive hormonal factors, among females only*							
Ever used hormonal replacement therapy	23,478 (35%)	14,317 (36%)		2961 (34%)	3067 (33%)	3133 (34%)	
Ever used oral contraceptives	57,613 (86%)	34,166 (86%)		7646 (87%)	8000 (86%)	7801 (85%)	
Number of pregnancies	2.0 (0.0, 4.0)	2.0 (0.0, 4.0)		2.0 (0.0, 4.0)	2.0 (0.0, 4.0)	2.0 (0.0, 4.0)	
*Serum bilirubin levels*							
Normal (< 21 µmol/L)	111,214 (91%)	66,045 (92%)		13,926 (91%)	16,155 (91%)	15,088 (91%)	
Elevated (≥ 21 µmol/L)	3240 (2.7%)	1884 (2.6%)		411 (2.7%)	496 (2.8%)	449 (2.7%)	
Unknown	7139 (5.9%)	4159 (5.8%)		925 (6.1%)	1,055 (6.0%)	1000 (6.0%)	
Related conditions^f^	15,909 (13%)	9380 (13%)		1975 (13%)	2379 (13%)	2175 (13%)	
Familial diabetes^g^	24,706 (20%)	14,366 (20%)		3,118 (20%)	3665 (21%)	3557 (22%)	
*Food group consumption* ^*a*^ *, g/week*							
Red and processed meat	372 (0, 845)	396 (0, 847)		372 (0, 840)	368 (0, 867)	280 (0, 840)	
Poultry	175 (0, 607)	228 (0, 607)		182 (0, 607)	152 (0, 607)	0 (0, 607)	
Fish	175 (0, 595)	175 (0, 607)		175 (0, 560)	175 (0, 574)	129 (0, 630)	
Refined cereals	851 (257, 1,677)	849 (254, 1,679)		873 (289, 1,649)	870 (273, 1,680)	818 (238, 1,687)	
Whole grain cereals	475 (0, 1,425)	434 (0, 1,385)		490 (0, 1,405)	518 (0, 1,468)	588 (0, 1,622)	
Mixed dishes	210 (0, 1,173)	175 (0, 1,146)		327 (0, 1,181)	263 (0, 1,208)	303 (0, 1,348)	
Dairy	1943 (718, 3456)	1986 (840, 3483)		1948 (782, 3399)	1986 (756, 3504)	1601 (292, 3290)	
Fats	80 (8, 181)	81 (8, 182)		75 (8, 172)	81 (8, 184)	77 (6, 183)	
Fruits	1339 (280, 2,902)	1288 (245, 2,814)		1365 (350, 2,901)	1383 (327, 2,989)	1507 (350, 3,206)	
Nuts	7 (0, 151)	0 (0, 140)		14 (0, 154)	11 (0, 165)	11 (0, 190)	
Vegetables	1189 (331, 2,569)	1126 (299, 2,421)		1242 (450, 2,538)	1267 (398, 2,748)	1348 (381, 3,060)	
Potatoes	624 (0, 1,260)	630 (0, 1,260)		613 (0, 1,248)	624 (0, 1,260)	630 (0, 1,321)	
Eggs and egg dishes	0 (0, 420)	0 (0, 420)		70 (0, 420)	88 (0, 438)	70 (0, 513)	
Non-alcoholic beverages	10,728 (7149, 15,295)	10,605 (770, 15,155)		10,789 (7268, 15,243)	10,815 (7280, 15,479)	11,060 (7333, 15,820)	
Alcoholic beverages	961 (0, 4,720)	992 (0, 4,835)		1,078 (0, 4,725)	963 (0, 4,835)	653 (0, 4288)	
Snacks and sweets	516 (112, 1,192)	525 (114, 1,211)		494 (117, 1,117)	511 (116, 1,176)	493 (98, 1,204)	
Sauces and condiments	117 (0, 385)	117 (0, 385)		127 (0, 385)	117 (0, 373)	105 (0, 390)	
Total weight of consumed foods	22,035 (16,616, 28,894)	21,703 (16,344, 28,409)		22,138 (16,761, 28,633)	22,457 (17,060, 29,523)	22,997 (17,374, 30,390)	

Continuous variables are presented as median (10%, 90%) and categorical values as number of participants (%). ^a^The definition and inclusion of foods in each food group can be found in supplementary Table 1. ^b^Educational level was defined as low (Certificate of secondary education (CSE), national vocational qualifications, higher national diploma, higher national certificates, other professional qualifications, or equivalent), intermediate (A levels, O levels, general certificate of secondary education, or equivalent), and high (College or University degree). ^c^Deprivation was assessed with the Townsend Deprivation Index based on four indicators of material deprivation: non-home ownership, non-car ownership, unemployment, and overcrowding. Positive values indicate that individuals live in areas with high material deprivation and negative values indicate relative affluence (25). ^d^Physical activity was based on total metabolic equivalent task (MET) minutes per week for all activity including walking, moderate, and vigorous activity, and defined as low (0–9.9 METs/week), moderate (10–49.9 METs/week), high (≥ 50 METs/week), and unknown. ^e^Participants’ self-reported weight change in the past year assessed through the question: "Compared with one year ago, has your weight changed? “. The answer: “Yes, I have lost weight” was used as indicator for recent weight loss. ^f^Related conditions cover participants’ diagnosis of diabetes, elevated cholesterol levels, hepatitis, or liver cirrhosis. ^g^Familial diabetes indicate diabetes diagnosis in participants’ biological mother, father, and/or sibling(s)

Replacing 80 g/week of red and processed meat with legumes was associated with a lower rate of GBD (Model 2; HR: 0.97, 95% CI 0.95; 0.98). However, replacing poultry or fish with legumes was not associated with GBD rate (HR poultry: 0.99, 95% CI 0.97; 1.00; HR fish: 1.00, 95% CI 0.98; 1.02). Adjusting for BMI did not change the magnitude or direction of associations (Table 2).

**Table 2:** Hazard ratios and 95% confidence intervals for gallbladder disease in the UK Biobank when replacing 80 g/week of meat, poultry, or fish with 80 g/week of legumes

Statistical model	Consuming 80 g/week of legumes instead of 80 g/week of		
Full sample (N = 121,593 events = 3772)	Red and processed meat^a^	Poultry^b^	Fish^c^
Model 1	0.98 (0.97; 0.99)	0.99 (0.98; 1.00)	1.01 (1.00; 1.03)
Model 2	0.97 (0.95; 0.98)	0.99 (0.97; 1.00)	1.00 (0.98; 1.02)
Model 3	0.98 (0.96; 0.99)	1.00 (0.98; 1.01)	1.00 (0.99; 1.02)
Legume consumers^d^(N = 49,505, events = 1477)			
Model 2	0.97 (0.95; 0.99)	0.99 (0.97; 1.01)	0.99 (0.97; 1.01)

Model 1 was stratified for age at recruitment, sex, and geographical region of recruitment, and adjusted for g/week intake of all other dietary components (red and processed meat, poultry, fish, refined cereal, whole grain cereal, fruits, vegetables, potatoes, nuts, dairy, fats, eggs and egg-dishes, mixed dishes, snacks and sweets, sauce and condiments, non-alcoholic beverages, and alcoholic beverages) apart from the food to be substituted, and total intake of all dietary components in g/week. Model 2 was further adjusted for ethnicity, Townsend Deprivation Iindex, educational level, yearly income, cohabitation, physical activity, smoking status, recent weight loss, history of gallbladder related conditions, serum bilirubin level, use of hormonal drugs for women, number of pregnancies for women, and family history of diabetes. Model 3 was further adjusted for BMI ≥ 30 kg/m^2^. ^a^Red and processed meat included beef, pork, lamb, and other meats including offal, and sausages, bacon, ham, and liver pâté. ^b^Poultry included poultry with or without skin and fried poultry with batter or breadcrumbs. ^c^Fish included oily fish, white fish, tinned tuna, fried fish with batter or breadcrumbs, and shellfish. ^d^Study sample after excluding participants who reported no consumption of legumes at either of the completed dietary assessments

### Secondary and sensitivity analyses

Excluding non-consumers of legumes, reducing the sample size to 49,505 participants (n events = 1477), did not change the associations, although the smaller sample size resulted in wider CIs (Table 2). An 80 g/week increase in legume consumption without substituting other elements of the diet was not associated with GBD (HR: 1.00, 95% CI 0.99; 1.01). Separating GBD into distinct outcomes or stratifying the analyses on age, sex, or BMI did not alter the associations markedly, however there was little evidence of an association in men (Supplementary Table 3).

Including peas or excluding soy milk from legume intake resulted in similar associations as the main analyses (Table 3). Excluding participants with unknown or high (≥ 90th percentile) bilirubin concentrations, fewer than three completed Oxford WebQs, or previous history of diabetes, elevated cholesterol, hepatitis, or cirrhosis slightly affected the width of the CIs without altering the magnitude or direction of the associations (Table 3). Separating red and processed meat indicated similar associations as the main analyses (HR red meat: 0.97, 95% CI 0.96; 0.99; HR processed meat: 0.96, 95% CI 0.94; 0.99).

**Table 3:** Hazard ratio and 95% confidence intervals for gallbladder disease when replacing 80 g/week of meat, poultry, or fish with 80 g/week of legumes across altered exposure level and exclusion criteria

	Hazard ratios and 95% confidence intervals		
Altered exposure level	Red and processed meat^a^	Poultry^b^	Fish^c^
Replacing 80 g/week of legumes and peas for animal-based foods, N = 121,593, events = 3772	0.97 (0.94; 1.00)	0.99 (0.96; 1.03)	1.00 (0.97; 1.04)
Replacing 80 g/week of legumes without soymilk for animal-based foods, N = 121,593, events = 3772	0.96 (0.94; 0.98)	0.98 (0.95; 1.00)	0.99 (0.97; 1.01)
Altered exclusion criteria			
< 90th percentile of bilirubin^d^, N = 103,002, events = 3184	0.97 (0.95; 0.98)	0.98 (0.97; 1.00)	1.00 (0.98; 1.02)
≥ 3 24 h recalls^e^, N = 75,456, events = 2287	0.97 (0.95; 0.98)	0.99 (0.97; 1.01)	1.00 (0.97; 1.02)
No previous history of related conditions^f^, N = 105,684, events = 3159	0.96 (0.95; 0.98)	0.99 (0.97; 1.00)	1.00 (0.98; 1.02)

Analyses followed adjustments in Model 2 and were stratified for age at recruitment, sex, and geographical region of recruitment, and adjusted for g/week intake of all other dietary components (red and processed meat, poultry, fish, refined cereal, whole grain cereal, fruits, vegetables, potatoes, nuts, dairy, fats, eggs and egg-dishes, mixed dishes, snacks and sweets, sauce and condiments, non-alcoholic beverages, and alcoholic beverages) apart from the food to be substituted, total intake of all dietary components in g/week, ethnicity, Townsend Deprivation Index, educational level, yearly income, cohabitation, physical activity, smoking status, recent weight loss, history of gallbladder related conditions, serum bilirubin level, use of hormonal drugs for women, number of pregnancies for women, and family history of diabetes. ^a^Red and processed meat included beef, pork, lamb, and other meats including offal, and sausages, bacon, ham, and liver pâté. ^b^Poultry included poultry with or without skin and fried poultry with batter or breadcrumbs. ^c^Fish included oily fish, white fish, tinned tuna, fried fish with batter or breadcrumbs, and shellfish. ^d^Analyses including individuals with alanine aminotransferase levels below 40 U/L. ^e^Analyses restricted to individuals with three or more completed 24-h dietary assessments. ^f^Analyses restricted to participants with no history of diabetes, elevated cholesterol, hepatitis, or cirrhosis

## Discussion

Replacing 80 g/week of red and processed meat with legumes was associated with a slightly lower rate of GBD in this large prospective cohort of British individuals, although evidence was stronger in women. Comparing legume consumption differing by 80 g/week without any food substitutions was not associated with GBD. While large proportions of GBD in Western countries relate to overweight and obesity [8], accounting for obesity in the analyses did not alter the associations.

Substitution analyses can provide important information on diet-disease relationships, directly supporting food-based dietary guidelines recommending replacing one type of food with another [19, 38, 39]. Although dietary guidelines usually specify serving sizes of foods in e.g. grams calculated relative to average adult caloric requirements, a recent study indicated that substitution studies including dietary variables in weight units as well as energy units may lead to spurious results [39]. We therefore chose to model all dietary variables in g/week, in line with the wording of current food-based dietary guidelines. The modest findings in our study suggest that for a population like that of the UK Biobank cohort, replacing one weekly serving of red and processed meat with legumes and pulses might lead to a lower risk of GBD over time or keep it stable. The serving size was chosen based on the UK National Health Service definition of a serving of fruits, vegetables and legumes [34]. Previous cohort studies investigating the association between legume consumption and GBD have not employed specified food substitution models accounting for total energy intake [18], meaning that people with differing legume consumption also have differing consumption of other calorie-providing foods [35]. Two prospective studies found a lower risk of cholecystectomy with higher consumption of legumes, but did not specify replacement foods [7, 40].

Dietary habits are considered modifiable risk factors for GBD with proposed mechanisms involving hepatobiliary lipid metabolism and cholesterol secretion through the gallbladder. A recent randomized controlled trial (RCT) comparing a legume-enriched calorie-restricted diet to a standard calorie-restricted control diet over 16 weeks found significantly greater reductions in total and LDL-cholesterol in the legume group compared to the control [41]. The suggested pathways involve the gut microbiome and the capacity of legume fibres and bile acids to bind lipids in the small intestine during digestion [41]. Similar findings have been reported in a meta-analysis of 10 RCTs, underscoring the significant role of legume fibre in lipid metabolism [42]. Legume fibre may influence bile acid regulation and inhibit lipid absorption in the small intestine, thereby reducing the pool of liver cholesterol available for secretion into the bile [42, 43]. This could prevent bile supersaturation with cholesterol and reduce gallstone formation, potentially lowering GBD risk [1, 4, 43]. Additionally, legume consumption appears to increase the abundance of fibre-degrading bacteria in the gut microbiome, which may improve the gut function though positive feedback loops involving improved bile acid regulation [41]. While our study did not directly address the underlying mechanisms, the lower GBD rate observed when legumes replaced red and processed meat suggests that legume fibre may play a key role [1, 41, 42]. The association could, however, also be driven by stimulated gallbladder contractions, the cholesterol-lowering effect of legume peptides, or the lower saturated fat intake due to lower meat intake [8, 44, 45]. The effect of legume fibre on GBD ultimately depends on the balance between these processes and other dietary and lifestyle factors [43, 46]. Replacing red and processed meat with legumes showed benefits for women, whereas no significant associations were found for other substitutions or among men. This difference could potentially be attributed to oestrogen promoting gallstone development through altered bile composition [47], or differences in how men and women metabolize legume components like phytoestrogens [48].

This study has several strengths including the prospective cohort design with a long follow-up period, large sample size, and detailed assessment of lifestyle and dietary intake. Furthermore, the food-level substitution analysis investigates the potential impact of replacing red and processed meat, poultry, or fish with legumes on the rate of GBD, thus providing a more clear interpretation. The sensitivity analyses underscore robustness of results. While having three or four assessments improves the estimation of dietary intake, including participants with two assessments still provides valuable data and has been shown to reasonably approximate habitual intake [24, 49–51]. Most participants completed two Oxford WebQs, and restricting analyses to those with three or more completed assessments did not change the direction or magnitude of associations, supporting prior evidence that two or three short-term dietary assessments yield comparable estimates of diet–disease associations [52]. The prospective study design reduced the risk of non-differential participation with regards to the outcome, and selection bias is therefore unlikely to have affected our results. However, this study also has some limitations. Dietary intake was assessed using self-reported 24 h dietary assessments, which are subject to random and systematic error. While using at least two dietary assessments helped to reduce random error and better estimate habitual intake, this may have introduced selection bias by excluding participants with fewer assessments, potentially enriching the sample for more health-conscious individuals. Nevertheless, results were robust to restricting analyses to those with three or more WebQs, suggesting that additional measurements had limited impact on the observed associations. A validation study of the Oxford WebQ identified person-specific biases in the correlation with true intake for certain nutrients, particularly among individuals with higher BMI [53]. Adjusting for BMI did not alter our results significantly. Despite the thorough outcome assessment, some GBD events may remain undiagnosed, as cholelithiasis may be asymptomatic. Although we adjusted for many confounding factors, residual confounding cannot be ruled out. We cannot explain the lack of association among men. The substitution analyses were furthermore purely observational, limiting any causal inference from the results. Future randomized controlled trials would be needed to confirm the findings of our study in a causal setting.

## Conclusion

Statistically replacing 80 g/week of red and processed meat with legumes was associated with a lower rate of GBD in this UK population, mainly in women. No association was observed when replacing poultry or fish with legumes. Further research in populations with higher legume intake or RCTs investigating underlying mechanisms are warranted to confirm these findings.

## Supplementary Information

Below is the link to the electronic supplementary material.

Supplementary Material 1

## Data Availability

The study was based on data from the UK Biobank prospective cohort that can be accessed through an application to the Access Management System of UK Biobank online (https://www.ukbiobank.ac.uk/enable-your-research/apply-for-access). Any discrepancies from the study protocol [54] have been explained. Analyses were structured to be reproducible using the targets R package [55]. Code is available online at https://github.com/steno-aarhus/lega/ (Accessed on 3 April 2025).
