# Supplementary material for: Replacing red and processed meat, poultry, or fish with legumes and the risk of gallbladder diseases in a large British cohort
Source: Eur J Nutr. 2025 Nov 12;64(8):318. doi: 10.1007/s00394-025-03828-1 (PMC12612017; doi:10.1007/s00394-025-03828-1)
Supplement: Supplementary file 1 — Supplementary Material 1 [file 394_2025_3828_MOESM1_ESM.pdf]

Supplementary information to: Substituting meat, poultry, and fish with legumes and risk of gallstones in a large prospective cohort

Fie Langmann[1], Daniel B. Ibsen[1,2], Luke W. Johnston[2], Aurora Perez-Cornago[3], Christina C. Dahm[1]

1. Aarhus University, Department of Public Health, Bartholins Allé 2, 8000 Aarhus C, Denmark.

2. Steno Diabetes Center Aarhus, Aarhus University Hospital, Palle Juul-Jensens Boulevard 11, 8200 Aarhus C, Denmark

3. European Commission, Joint Research Centre (JRC), Ispra, Italy

Corresponding author:

Christina C. Dahm. Department of Public Health, Aarhus University, Bartholins Allé 2, 8000 Aarhus C, Denmark. E-mail: ccd@ph.au.dk, phone: +45 2332 1875

Contents

Supplementary Table 1. 3
Supplementary Table 2. 5
Supplementary Table 3. 9
Supplementary Figure 1. 10
References 11

Supplementary Table 1. Description of dietary components included in the definition of food groups for the study.

Food group	Dietary components
Legumes	Legumes and pulses, soy desserts and yoghurt, soy milk, meat substitutes made from soy.
Red and processed meat	Beef, pork, lamb, offal, sausages, bacon, ham, and liver pâté.
Poultry	Poultry with or without skin and fried poultry with batter or breadcrumbs.
Fish	Oily fish, white fish, tinned tuna, fried fish with batter or breadcrumbs, and shellfish.
Eggs and egg dishes	Eggs and egg-based dishes like scrambled eggs, omelets, and quiche.
Refined cereal	White pasta and rice, white bread, biscuit cereal, biscuits, savory crackers, mixed bread (50/50 brown and seeded), other bread, and other cereal with high sugar content.
Whole grain cereal	Whole meal bread, whole meal pasta, brown rice, bran cereal, oat cereal (with and without added sugar), muesli, and other whole grains.
Dairy	Milk (whole, semi-skimmed, skimmed), cream, cheese (low, medium, and high fat), yoghurt (low or full fat), milk-based and powdered drinks, and milk-dairy desserts.
Fruits	Berries, citrus fruits, apples, pears, dried fruits, stewed fruits, and other fruits.
Vegetables	Green leafy vegetables, cabbages, onion, garlic, root vegetables, raw salad, peas, sweetcorn, vegetable dips (guacamole and hummus), tomatoes, mushrooms, mixed vegetables, and vegetable side dishes.
Potatoes	Fried/roasted potatoes, mashed potatoes, and baked or boiled potatoes and sweet potatoes.
Nuts	Salted and unsalted nuts and seeds.
Fats	Olive oil, animal-based fat spreads, and plant-based fat spreads.

Mixed dishes	Pizza, grain dishes with added fat, samosa, pakora, soups, sushi, and vegetarian non-soy meat substitutes like quorn.
Sauce and condiments	Sauces and condiments with low or high fat content.
Snacks and sweets	Nut-based spreads, savoury snacks, chocolate confectionery, added sugars and preserves, cakes, pastry, other desserts, and other sweets.
Non-alcoholic beverages	Coffee, tea, rice milk, oat milk, water (still or sparkling), sugar-sweetened soft drinks, artificially sweetened soft drinks, and fruit juices.
Alcoholic beverages	Beer, cider, spirits, and fortified and non-fortified wines.

Supplementary Table 2. Baseline characteristics of participants in the UK Biobank cohort across incident gallbladder disease (N = 121,593)

Characteristics	**All participants**<br>N = 121,593	**With GBD[a]**<br>N = 3,772
**Sex, female**	67,291 (55%)	2,245 (60%)
**Age, years**	57.0 (44.0, 66.0)	59.0 (46.0, 67.0)
**Yearly income £**		
>100,000	8,808 (7.2%)	145 (3.8%)
52,000-100,000	28,353 (23%)	638 (17%)
31,000-51,999	32,116 (26%)	991 (26%)
18,000-30,999	26,147 (22%)	933 (25%)
<18,000	15,089 (12%)	647 (17%)
Unknown	11,080 (9.1%)	418 (11%)
**Educational level[b]**		
High	57,341 (47%)	1,385 (37%)
Intermediate	40,409 (33%)	1,385 (37%)
Low	23,843 (20%)	1,002 (27%)
**Deprivation[c]**	-2.4 (-4.7, 2.6)	-2.3 (-4.5, 2.7)
**Cohabitation**		
Alone	21,654 (18%)	684 (18%)
With spouse/partner	52,718 (43%)	1,812 (48%)
Other non-partner	47,019 (39%)	1,268 (34%)
Unknown	202 (0.2%)	8 (0.2%)
**Ethnicity**		
White	117,375 (97%)	3,663 (97%)
Other	4,218 (3.5%)	109 (2.9%)
**Physical activity[d]**		
High	21,233 (17%)	577 (15%)
Moderate	53,952 (44%)	1,442 (38%)
Low	29,404 (24%)	1,091 (29%)
Unknown	17,004 (14%)	662 (18%)
**Smoking status**		

Current, >15 cigarettes/day	1,756 (1.4%)	74 (2.0%)
Current, <15 cigarettes/day	3,304 (2.7%)	107 (2.8%)
Former	43,410 (36%)	1,505 (40%)
Never	69,498 (57%)	1,987 (53%)
Unknown	3,625 (3.0%)	99 (2.6%)
**Anthropometry**		
Weight loss past year[e]	18,310 (15%)	707 (19%)
BMI ≥ 30 kg/m^2	23,583 (19%)	1,320 (35%)
**UK-region of recruitment**		
East Midlands	9,727 (8.0%)	317 (8.4%)
London	25,085 (21%)	671 (18%)
North East	11,469 (9.4%)	409 (11%)
North West	14,904 (12%)	546 (14%)
Scotland	6,362 (5.2%)	149 (4.0%)
South East	11,458 (9.4%)	317 (8.4%)
South West	12,668 (10%)	367 (9.7%)
Wales	3,783 (3.1%)	70 (1.9%)
West Midlands	7,374 (6.1%)	251 (6.7%)
Yorkshire and Humber	18,763 (15%)	675 (18%)
**Female reproductive hormonal factors, among females only**		
Ever used hormonal replacement therapy	23,478 (35%)	979 (44%)
Ever used oral contraceptives	57,613 (86%)	1,908 (85%)
Number of pregnancies	2.0 (0.0, 4.0)	2.0 (0.0, 4.0)
**Serum bilirubin levels[f]**		
Normal (< 21 µmol/L)	111,214 (91%)	3,442 (91%)
Elevated (≥ 21 µmol/L)	3,240 (2.7%)	110 (2.9%)
Unknown	7,139 (5.9%)	220 (5.8%)
**Related conditions[g]**	15,909 (13%)	613 (16%)

**Familial diabetes[h]**	24,706 (20%)	931 (25%)
**Food group consumption[i], g/week**		
Legume consumption	0 (0, 473)	0 (0, 473)
Red and processed meat	372 (0, 845)	420 (0, 886)
Poultry	175 (0, 607)	182 (0, 607)
Fish	175 (0, 595)	140 (0, 560)
Refined cereals	851 (257, 1,677)	839 (259, 1,645)
Whole grain cereals	475 (0, 1,425)	409 (0, 1,353)
Mixed dishes	210 (0, 1,173)	210 (0, 1,155)
Dairy	1,943 (718, 3,456)	1,968 (735, 3,477)
Fats	80 (8, 181)	81 (8, 182)
Fruits	1,339 (280, 2,902)	1,321 (233, 2,948)
Nuts	7 (0, 151)	0 (0, 140)
Vegetables	1,189 (331, 2,569)	1,138 (286, 2,615)
Potatoes	624 (0, 1,260)	630 (0, 1,307)
Eggs and egg dishes	0 (0, 420)	0 (0, 420)
Non-alcoholic beverages	10,728 (7,149, 15,295)	10,695 (7,105, 15,248)
Alcoholic beverages	961 (0, 4,720)	613 (0, 4,018)
Snacks and sweets	516 (112, 1,192)	558 (123, 1,260)
Sauces and condiments	117 (0, 385)	117 (0, 368)
Total weight of consumed foods	22,035 (16,616, 28,894)	21,740 (16,164, 28,614)

Continuous variables are presented as median (10%, 90%) and categorical values as number of participants (%). [a]GBD, gallbladder disease. [b]Educational level was defined as low (Certificate of Secondary Education (CSE), National Vocational Qualifications, Higher National Diploma, Higher National Certificates, other professional qualifications, or equivalent), intermediate (A levels, O levels, General Certificate of Secondary Education, or equivalent), and high (College or University degree). [c]Deprivation was assessed with the Townsend Deprivation Index based on four indicators of material deprivation: non-home ownership, non-car ownership, unemployment, and overcrowding. Positive values indicate that individuals live in areas with high material deprivation and negative values indicate relative affluence [1]. [d]Physical activity was based on total metabolic equivalent task (MET) minutes per week for all activity including walking, moderate, and vigorous activity, and defined as low (0-9.9 METs/week), moderate (10-49.9 METs/week), high (≥50 METs/week), and unknown. [e]Participants' self-reported weight change in the past year assessed through the question: "Compared with one year ago, has your weight changed?". The answer: "Yes, I have lost weight" was used as indicator for recent

weight loss. [f]The cut-off levels Serum bilirubin levels were defined based on National Health Services cut-offs as normal: < 21 µmol/L, elevated: ≥ 21 µmol/L, or unknown [2]. [g]Related conditions cover participants' diagnosis of diabetes, elevated cholesterol levels, hepatitis, or liver cirrhosis. [h]Familial diabetes indicate diabetes diagnosis in participants' biological mother, father, and/or sibling(s). [i]See Supplementary Table 1 for definitions of each food group .

Supplementary Table 3. Hazard ratios and 95 % confidence intervals for gallbladder disease in the UK Biobank when replacing 80 g/week of meat, poultry, or fish with 80 g/week of legumes across strata of gallbladder disease, sex, age, or BMI

	Hazard ratios and 95 % confidence intervals		
**Strata**	Red and processed meat[a]	Poultry[b]	Fish[c]
**Gallbladder disease, N = 121,593**			
Cholelithiasis, n events = 3301	0.97 (0.95; 0.98)	0.98 (0.97; 1.00)	1.00 (0.98; 1.02)
Cholecystectomy, n events = 2257	0.97 (0.95; 0.98)	0.98 (0.96; 1.00)	1.00 (0.98; 1.02)
Cholecystit, n events = 574	0.98 (0.95; 1.02)	1.00 (0.96; 1.04)	1.04 (0.99: 1.09)
**Sex[d]**			
Male, N = 54,302, events = 1527	1.00 (0.98; 1.02)	1.00 (0.98; 1.02)	1.02 (1.00; 1.04)
Female, N = 67,291, events = 2245	0.97 (0.96; 0.98)	0.99 (0.97; 1.00)	1.00 (0.98; 1.02)
**Age[e], years**			
< 53, N = 40,531, events = 944	0.97 (0.95; 1.00)	0.99 (0.96; 1.02)	0.99 (0.96; 1.02)
53-61, N = 40, 531, events = 1286	0.97 (0.94; 0.99)	0.99 (0.96; 1.01)	1.01 (0.98; 1.04)
> 61, N = 40,531, events = 1542	0.97 (0.95; 1.00)	0.98 (0.95; 1.01)	1.01 (0.98; 1.04)
**BMI, kg/m^2**			
< 25, N = 47,884, events = 850	0.99 (0.97; 1.02)	1.01 (0.98; 1.04)	1.02 (0.98; 1.05)
25-29.9, N = 50126, events = 1602	0.98 (0.95; 1.00)	1.00 (0.97; 1.03)	1.00 (0.97; 1.02)
≥ 30, N = 98,010, events = 2452	0.98 (0.96; 0.99)	1.00 (0.98; 1.02)	1.00 (0.98; 1.02)

Analyses followed adjustments of Model 2 and was stratified for age at recruitment, sex, and geographical region of recruitment, and adjusted for g/week intake of all other dietary components (red and processed meat, poultry, fish, refined cereal, whole grain cereal, fruits, vegetables, potatoes, nuts, dairy, fats, eggs and egg-dishes, mixed dishes, snacks and sweets, sauce and condiments, non-alcoholic beverages, and alcoholic beverages) apart from the food to be substituted, total intake of all dietary components in g/week, ethnicity, Townsend Deprivation Index, educational level, yearly income, cohabitation, physical activity, smoking status, recent weight loss, history of gallbladder related conditions, serum bilirubin level, use of hormonal drugs for women, number of pregnancies for women, and family history of diabetes. [a]Red and processed meat included beef, pork, lamb, and other meats including offal, and sausages, bacon, ham, and liver pâté. [b]Poultry included poultry with or without skin and fried poultry with batter or breadcrumbs. [c]Fish included oily fish, white fish, tinned tuna, fried fish with batter or breadcrumbs, and shellfish. [d]Substitution analyses not adjusted for sex-strata. [e]Substitution analyses not adjusted for age-strata at recruitment.

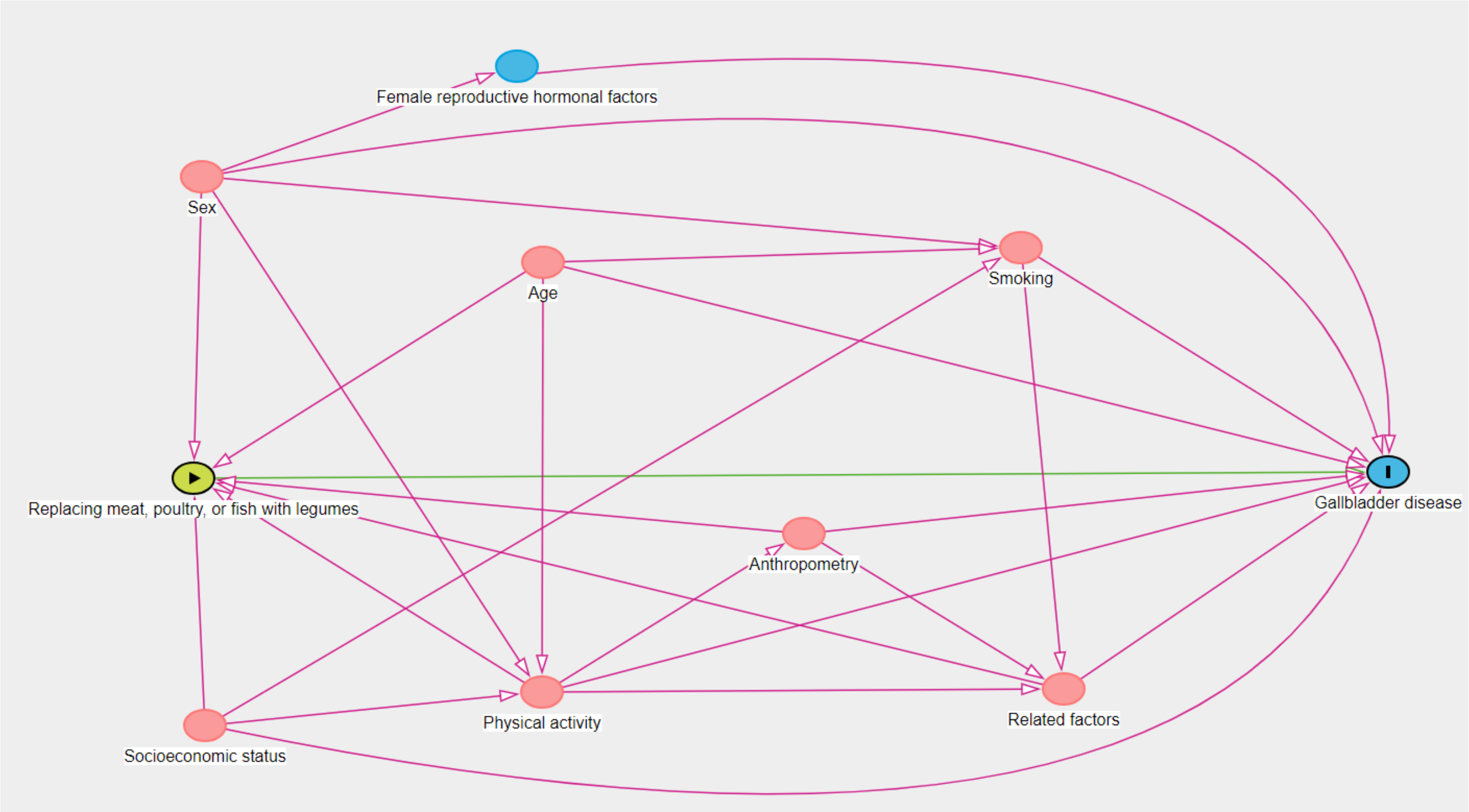

Supplementary Figure 1. Directed Acyclic Graph (DAG) representing the association between replacing red and processed meat, poultry, or fish for legumes and risk of gallbladder disease (cholecystitis, cholelithiasis, cholecystectomy) in the UK Biobank cohort. This DAG was made using the DAGitty software [3]. Female reproductive hormonal factors include use of hormonal replacement therapy drugs, use of oral contraceptives, and number of pregnancies. Socioeconomic status comprises factors like ethnicity, Townsend Deprivation Index, geographical living area, cohabitation status, educational level, and yearly income. Related factors include gallbladder related conditions such as serum bilirubin levels, diabetes, elevated cholesterol levels, hepatitis, or liver cirrhosis, or family history of diabetes. Anthropometry includes elevated BMI and rapid weight loss. exposure, outcome, ancestor of outcome, ancestor of exposure and outcome, causal path, biasing path

References

1. National Centre for Research Methods, (n.d.). *Geographical Referencing Learning Resources - Townsend Deprivation Index*. https://www.restore.ac.uk/geo-refer/36229dtuks00y19810000.php Accessed 21 October 2024
2. North Bristol NHS Trust, (2024). *Bilirubin*. https://www.nbt.nhs.uk/severn-pathology/requesting/test-information/bilirubin Accessed 4 October 2024
3. Textor, J., van der Zander, B., Gilthorpe, M. K., Liskiewicz, M., Ellison, G. T. H., (2016). *Robust causal inference using directed acyclic graphs: the R package 'dagitty'*. International Journal of Epidemiology. **45**(6): p. 1887-1894.https://doi.org/10.1093/ije/dyw341
